# Kinetics of dengue viremia and its association with disease severity: an ambispective study

**DOI:** 10.1007/s13337-024-00872-z

**Published:** 2024-06-29

**Authors:** Puneet Bhatt, Anup Jayaram, Muralidhar Varma, Chiranjay Mukhopadhyay

**Affiliations:** 1 Manipal Institute of Virology, Manipal Academy of Higher Education, Manipal, Karnataka 576104 India; 2 Department of Infectious Diseases, Kasturba Medical College, Manipal Academy of Higher Education, Manipal, Karnataka 576101 India

**Keywords:** Dengue, Dengue viremia (viral load), Dengue with warning signs (DWS), Dengue without warning signs (DwoWS), Severe dengue, Dengue hemorrhagic fever, Dengue shock syndrome

## Abstract

**Background:**

Dengue virus (DENV) infection is an important public health problem and causes significant morbidity and mortality. DENV typically causes a febrile illness that ranges from mild asymptomatic infection to fatal dengue hemorrhagic fever (DHF) and/or dengue shock syndrome (DSS). Early prediction of severe dengue disease is of utmost importance for providing prompt monitoring and treatment. The search for an ideal biomarker (host or viral factors) for early prediction of severe dengue remains elusive.

**Aim:**

To standardize a real time qRT-PCR for quantifying dengue viremia in serum samples and evaluate the kinetics of dengue viremia and its significance in disease severity.

**Results:**

In this ambispective study of 126 laboratory confirmed dengue patients, 72 were primary infections and 54 were secondary infections. The most common serotype was serotype 1 (n = 37) followed by serotype 2 (n = 34). According to WHO 1997 dengue case classification, 111 patients were cases of dengue fever (DF), 13 from DHF and 02 from DSS. Day 3 viremia levels were significantly elevated in severe dengue patients (DHF/DSS) as compared to that of DF (*p* < 0.05). However, no such association was found between viremia levels and serotype or immune status.

**Conclusion:**

Dengue viremia has a significant association with disease severity and day 3 viremia levels may be used as a predictor for dengue disease severity.

## Introduction

Dengue virus (DENV) infection is health concern for more than 100 countries, mainly in tropics and sub-tropics. It is estimated that approximately 4 billion people are at risk of acquiring the infection, with around 96 million manifesting clinically with different levels of severity [2, 10].

Dengue virus infection is a mosquito borne flavivirus infection, transmitted by mosquitos of the genus *Aedes,* predominantly *Aedes aegypti* [8, 10]. The disease has a wide spectrum of manifestations. It ranges from asymptomatic infection to moderate febrile illness (Dengue Fever) to more severe disease forms like Dengue Haemorrhagic Fever (DHF) and Dengue Shock Syndrome (DSS) [3].

World Health Organization (WHO) devised a formal classification scheme in 1997 that classified dengue into Dengue fever (DF), Dengue hemorrhagic fever (DHF grades 1 and 2) and Dengue shock syndrome (DHF grade 3 and 4) [7, 15]. The major problem with this classification system was that many cases with some warning signs were not able to be classified as DHF due to its strict clinical criteria. In 2009, WHO adopted the revised case classification for dengue with three levels of severity as having dengue without warning signs (DwoWS), dengue with warning signs (DWS), and severe dengue (SD) based on clinical manifestations with or without laboratory parameters. This system is considered better from the clinical point of view as it classifies patients with warning signs separately. These patients need more careful monitoring so that they do not deteriorate to severe disease, or even if they so appropriate and timely treatment can be administered. [12]. Both these classification systems and case definitions are used in clinical practice till date.

Many in-vitro and in-vivo studies have been done across the globe to ascertain the role of dengue viremia in the pathogenesis and severity of the disease. However, the results have been inconsistent. Moreover, there is dearth of literature regarding the dengue viremia kinetics and the association of dengue viremia with disease severity from the Indian context.

In view of this, this present study was carried out with an aim to evaluate the dengue viremia kinetics in human serum samples and the association of viral load with dengue disease severity.

## Material and methods

This study was carried out from May 2019 to April 2021 in a virology institute in Southern India, with patients included from the affiliated tertiary care hospital.

Patients included in the study were followed up from admission till discharge from the hospital. Daily blood samples were collected till the discharge of the patient. The clinical and other laboratory parameters were also recorded. Institutional ethics committee approval was taken before commencement of the study. Written informed consent was obtained from the patients in the prospective cohort.

*Inclusion criteria*: Hospitalized adults (age ≥ 18 years) with acute febrile illness (AFI), presented within 3 days of onset of fever with any other symptoms suggestive of dengue fever, like headache, body ache, retro-orbital pain, nausea/vomiting, petechiae/ecchymoses/purpura, bleeding gums, rash, abdominal pain, lethargy/restlessness

*Exclusion criteria*: Not willing to provide informed consent and without a legally authorized representative available; Immuno-compromised cases like HIV+ve (with CD4 count < 350/mm^3^), patients on chemotherapy or steroids or immunosuppressive drugs in last 1-year, post-transplant recipients; Known cases of Liver cirrhosis; Mixed infections (malaria, influenza, chikungunya, scrub typhus, leptospirosis, etc.)

In addition to this, some patient samples of laboratory confirmed dengue archived in our laboratory were included in the study retrospectively, which satisfied the following criteria: sample collection on Day 3 of symptoms onset, and age ≥ 18 years. The clinical and laboratory parameters of these patients were obtained by accessing the medical documents from the medical records department, after getting approval from Institutional ethics committee.

### Diagnosis of dengue virus infection

Laboratory confirmation of DENV infection was done by any one of the following:- DENV NS1 antigen ELISA (SD Bioline/Panbio), and/or IgM (MAC) ELISA (SD Bioline/Panbio).

### Classification of cases into primary or secondary dengue

Dengue IgG Capture ELISA (Panbio Diagnostics, Abbott, USA) was performed according to manufacturer’s instructions and was used in conjunction with the Panbio Dengue IgM Capture ELISA and Dengue Early ELISA (Panbio Diagnostics, Abbott, USA). In patients with either dengue NS1 positivity and/or IgM positivity along with high IgG levels indicate secondary dengue infection and can be detected as early as 3 days post onset of illness till 15 days. A positive result (> 22 Panbio units) in Panbio dengue IgG capture ELISA is indicative of active secondary infection [11].

### Serotyping of dengue

Total RNA extraction was done by QIAamp Viral RNA Mini Kit (QIAGEN). Conventional nested PCR was performed using primers described by Lanciotti RS et al. [9]. The PCR reaction mix of the two step nested PCR and the cycling conditions have been shown in Table 1.

**Table 1:** PCR mixture components and cycling conditions for serotyping of dengue virus by nested PCR

Step 1		Step 2	
NFW (Nuclease free water)	10.3 µl	NFW (Nuclease free water)	16.11 µl
MM (Master mix)	8.5 µl	MM (Master mix)	4.0 µl
D_1_	0.5 µl	D_1_	1.0 µl
D_2_	0.5 µl	TS1	1.0 µl
EM	0.2 µl	TS2	0.5 µl
Template	5 µl	TS3	0.312 µl
Total	25 µl	TS4	0.625 µl
		EM	0.2 µl
		Template (diluted 1:25 from step 1)	1.25 µl
		Total	25 µl
42 °C—60 min		94 °C—03 min	
94 °C—02 min			
94 °C—30 s	35 cycles	94 °C—30 s	25 cycles
55 °C—01 min		52 °C—01 min	
72 °C—01 min		72 °C—01 min	
72 °C—05 min		72 °C—05 min	
4 °C—∞		4 °C—∞	

### Viral load by real time quantitative reverse transcriptase PCR (qRT-PCR)

Real time quantitative reverse transcriptase PCT (Real time qRT-PCR) targeting the untranslated region (UTR) of DENV, a highly conserved region, was performed for determining the viral load in serum samples of dengue patients. The nucleotide sequences of the calibrator, primers and probe sequences were used as described by Gurukumar et al. [6] and are given in Table 2. The reaction was performed in 25 $\upmu$L volume using AgPath-ID™ One Step RT-PCR kit (Thermo Fisher Scientific, USA). The RT-PCR cycling conditions used were: reverse transcription at 50 °C for 30 min, denaturation at 95 °C for 15 min and 40 cycles amplification of 95 °C for 15 s and 58 °C for 30 s.

**Table 2:** Nucleotide sequence of primers, probe and standard calibrator used in the qRT-PCR assay

Primer	Sequence	Nucleotide position
Forward primer	5′-GARAGACCAGAGATCCTGCTGTCT-3′	10,635–10658
Reverse primer	5′-ACCATTCCATTTTCTGGCGTT-3′	10,708–10682
TaqMan MGB probe	5′-AGCATCATTCCAGGCAC-3′	10,663–10679
Dengue standard calibrator	5′-GAGAGACCAGAGATCCTGCTGT CTCTACAGCATCATTCCAGGCACA GAACGCCAGAAAATGGAATGGT-3′	–

The concentration (copies/$\upmu$L) of calibrator was calculated and a total of 15 dilutions were prepared and tested in triplicates to obtain the limit of detection and to determine which concentrations need to be used in the qRT-PCR for generating standard curve for viral load estimation. Based on the cycle threshold (Ct) values, it was decided that the dilutions from 10^–8^ to 10^–13^ will be run in triplicate in each RT-PCR run. This helped in obtaining an accurate standard curve graph and hence the precision to calculate the viral load in samples. The copy numbers of the dengue virus in the serum samples were log transformed and finally expressed as Log_10_copies/ml. The samples which did not show any viral copy numbers (below detection limit) were assigned an arbitrary value of 01copy/ml for the purpose of statistical analysis.

Peak viremia was defined as the highest value of viremia observed on a particular day in the illness. Time to viral clearance was described as the day of illness on which RT-PCR first became negative and remained negative thereafter.

### Statistical analysis

Statistical analysis of the data was carried out using ‘R’ version 3.6.1. Graphs were generated with the GraphPad Prism software version 5. Mann Whitney and Kruskal Wallis test was performed to assess the association of dengue viremia with disease severity, serotype, and immune status. Correlation coefficient was calculated for correlation between viral load and median platelet count and viral load and maximum percentage increase in hematocrit. Receiver operating characteristic (ROC) curve was constructed to assess the diagnostic ability of Day 3 viremia as a predictor of severe dengue disease. A *p* value of < 0.05 was considered as statistically significant.

## Results

### Study population

From May 2019 to April 2021, a total of 66 laboratory confirmed dengue in-patient cases were enrolled in the study and were followed up till discharge from the hospital. Daily blood samples were collected from the day of diagnosis till discharge. Out of these 66 patients, 6 were lost to follow-up. In addition to this, 66 patients of laboratory confirmed dengue were enrolled retrospectively whose first sample was collected on day 3 of symptom onset. So, a total of 126 patients of dengue were included in this study.

Out of 126 patients, 93 were males and 33 were females (male: female ratio 2.82). The age of the patients ranged from 18 to 80 years with mean age of 34.2 ± 15.4 years.

A total of 72 patients were found to be suffering from primary dengue and 54 from secondary dengue. Serotyping of dengue was performed by conventional nested RT-PCR. The most common serotype detected was Serotype-1 in 37 cases followed by serotype-2 in 34, serotype-3 in 10 and serotype-4 in 3 patients. However, in 42 cases, the serotype could not be detected.

The severity of dengue disease was classified based on the WHO case classification 1997 and revised classification 2009. According to the WHO 1997 case classification, 111 patients were suffering from dengue fever, 13 from DHF and 2 from DSS. As per the WHO revised 2009 case classification, 93 cases were dengue without warning signs (DwoWS), 20 were dengue with warning signs (DWS) and 13 were severe dengue (SD). The distribution of all cases according to immune status, serotype and disease severity are shown in Table 3.

**Table 3:** Immune status, serotype and severity of dengue cases

	WHO 1997 case classification			WHO 2009 revised classification			
	DF	DHF	DSS	DwoWS	DWS	SD	
Serotype	1 (37)	31	5	1	25	7	5
	2 (34)	27	6	1	20	7	7
	3 (10)	10	0	0	9	1	0
	4 (3)	3	0	0	3	0	0
	Not detected (42)	40	2	0	36	5	1
Immune status	Primary (72)	71	1	0	65	6	1
	Secondary (54)	40	12	2	28	14	12

### Viremia kinetics

**Fig. 1:**
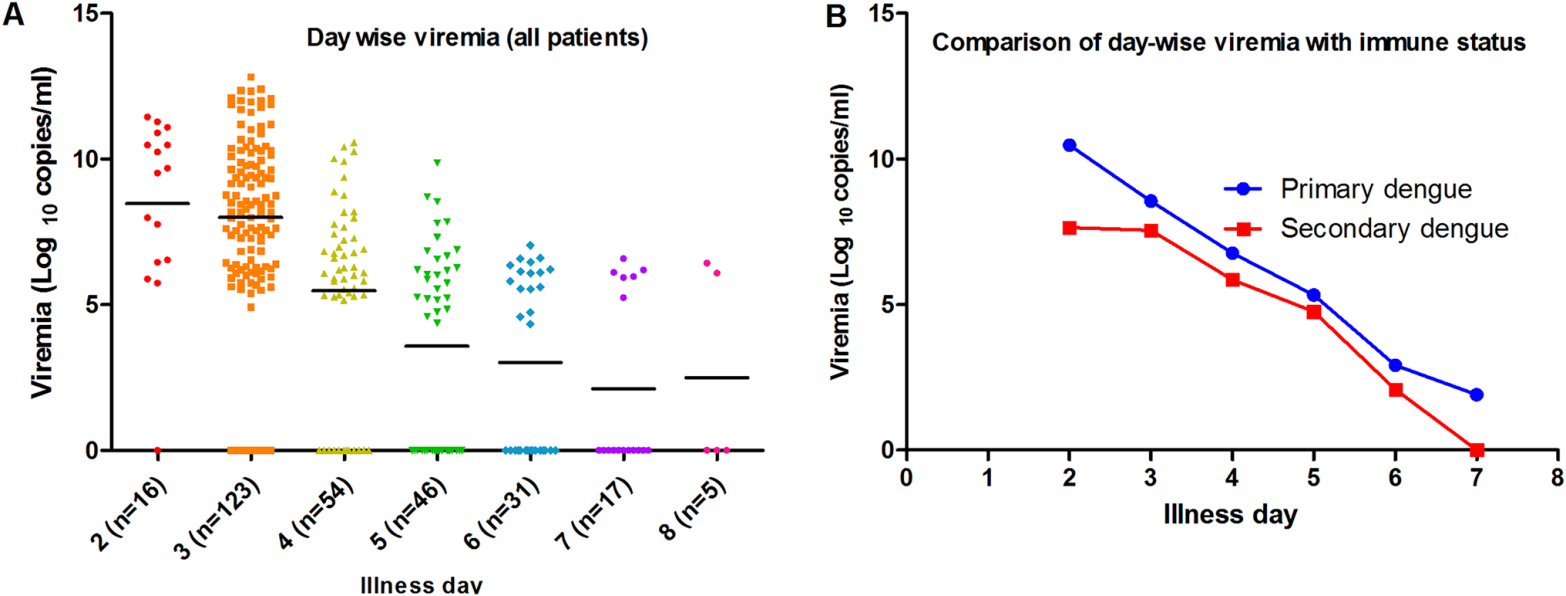
Day-wise dengue viremia levels. Each dot represents a sample and the horizontal black line represents the median. **A** Day-wise dengue viremia levels of all laboratory confirmed dengue patients. **B** Day-wise dengue viremia levels in primary and secondary dengue

Real time qRT-PCR was performed for quantification of viremia in daily serial samples of 60 prospective cases and Day 3 samples of 66 retrospective cases. The limit of detection of the assay was 40 copies/5 µL. The viremia levels were converted to copies/ml and then log-transformed to be expressed as log_10_ copies/ml. The viremia levels of all patients were plotted according to the illness days, and it was observed that the highest median viremia was seen on Day 2 (9.60 log_10_ copies/ml; IQR 6.48–10.8) followed by Day 3 (8.3 log_10_ copies/ml; IQR 6.32–10.1) as shown in Figure 1A. Viremia levels were seen to decrease as the dengue infection progressed.

### Viremia and immune status

**Fig. 2:**
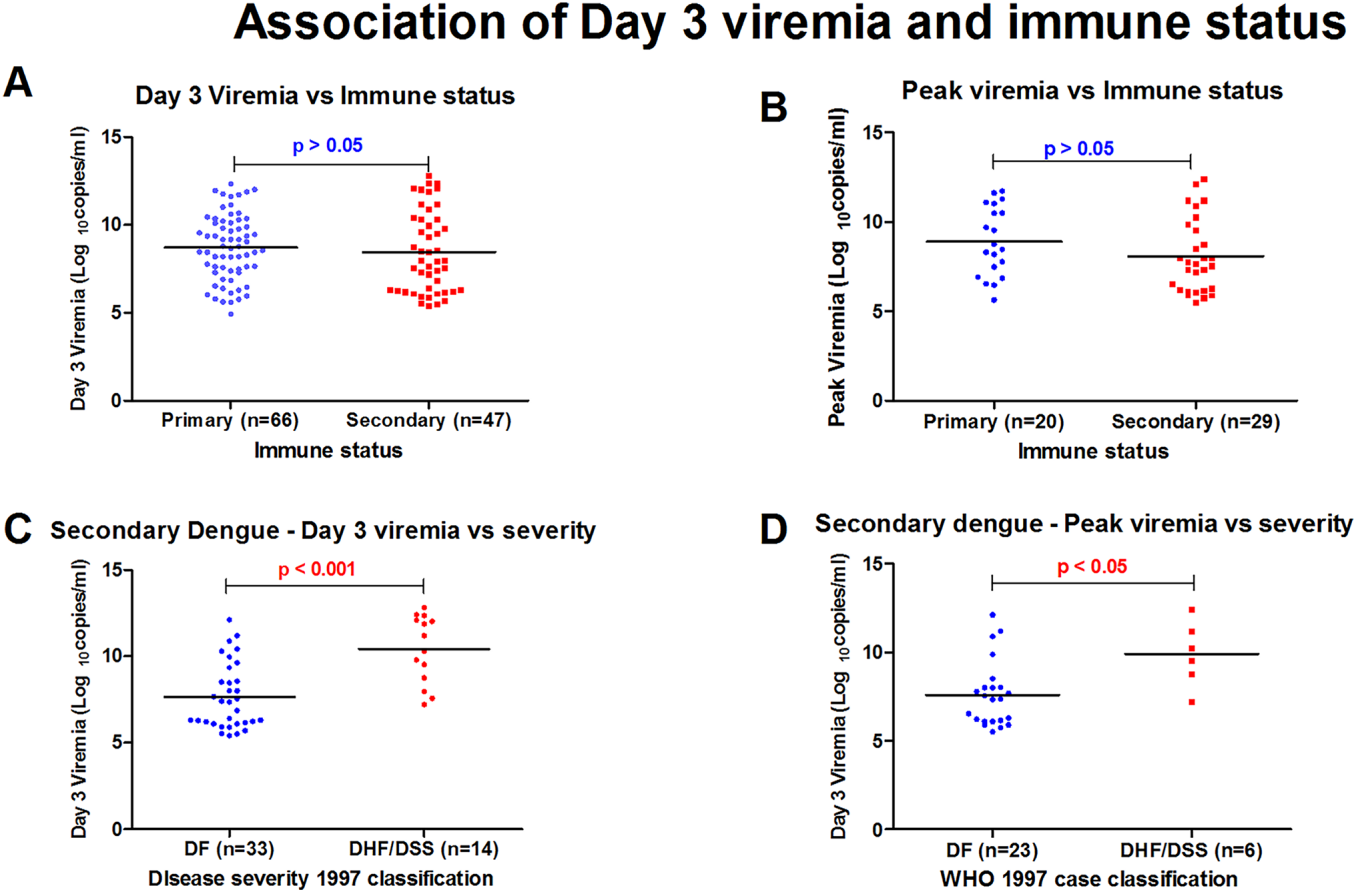
Association of dengue viremia with immune status. **A** Association of day 3 viremia with immune status. **B** Association of peak viremia with immune status. **C** Association of day 3 viremia with immune status in secondary dengue. **D** Association of peak viremia with immune status in secondary dengue

Day wise viremia was observed to be higher in primary dengue as compared to secondary dengue (Fig. 1B). The median Day 3 viremia was observed to be lower in secondary dengue (7.99 log_10_ copies/ml; IQR 6.28–10.31) as compared to primary dengue (8.72 log_10_ copies/ml; IQR 7.37–10.16). Similarly, peak viremia was also found to be lower in secondary dengue (7.67 log_10_ copies/ml; IQR 6.18–9.7) when compared to primary dengue (8.6 log_10_ copies/ml; IQR 7.05–10.89). However, none of these associations were found to be statistically significant (Fig. 2A and B).

In secondary dengue, median day 3 viremia was significantly higher in DHF/DSS patients (10.75 log_10_ copies/ml; IQR 8.56–12.16) as compared to DF patients (7.34 log_10_ copies/ml; IQR 6.13–8.95) and is shown in Figure 2C. Similarly, peak viremia was also observed to be significantly higher in DHF/DSS cases in secondary dengue cases (Fig. 2D).

Out of 54 secondary dengue cases, 14 were found to be DHF/DSS as compared to just one case of severe dengue in primary dengue (*p* < 0.01).

### Viremia and dengue serotype

Day wise median viremia levels were compared between serotype 1 and serotype 2 and no significant difference was found. Moreover, no statistically significant association was observed between day 3 median viremia or peak viremia and dengue serotypes. Also, no statistically significant difference was found between time to viral clearance and dengue serotypes.

### Viremia and disease severity

**Fig. 3:**
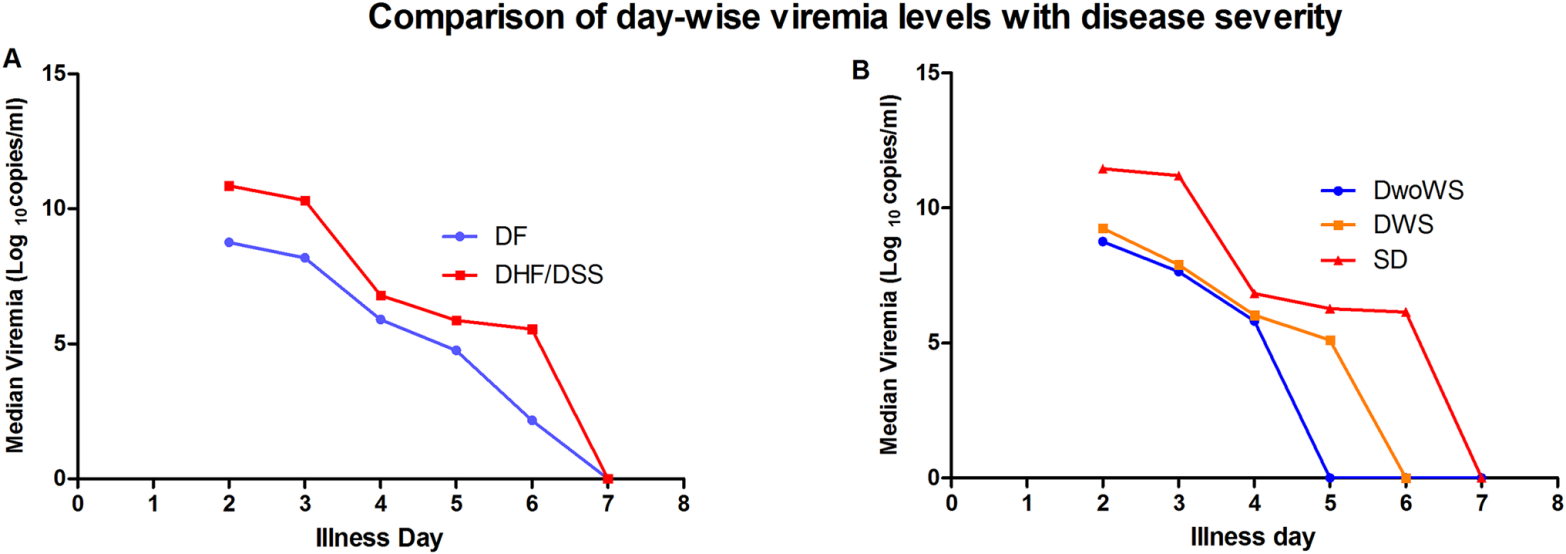
Comparison of day-wise viremia levels with disease severity. **A** Day-wise viremia levels in WHO 1997 dengue case classification. **B** Day-wise viremia levels in WHO 2009 revised dengue case classification

**Fig. 4:**
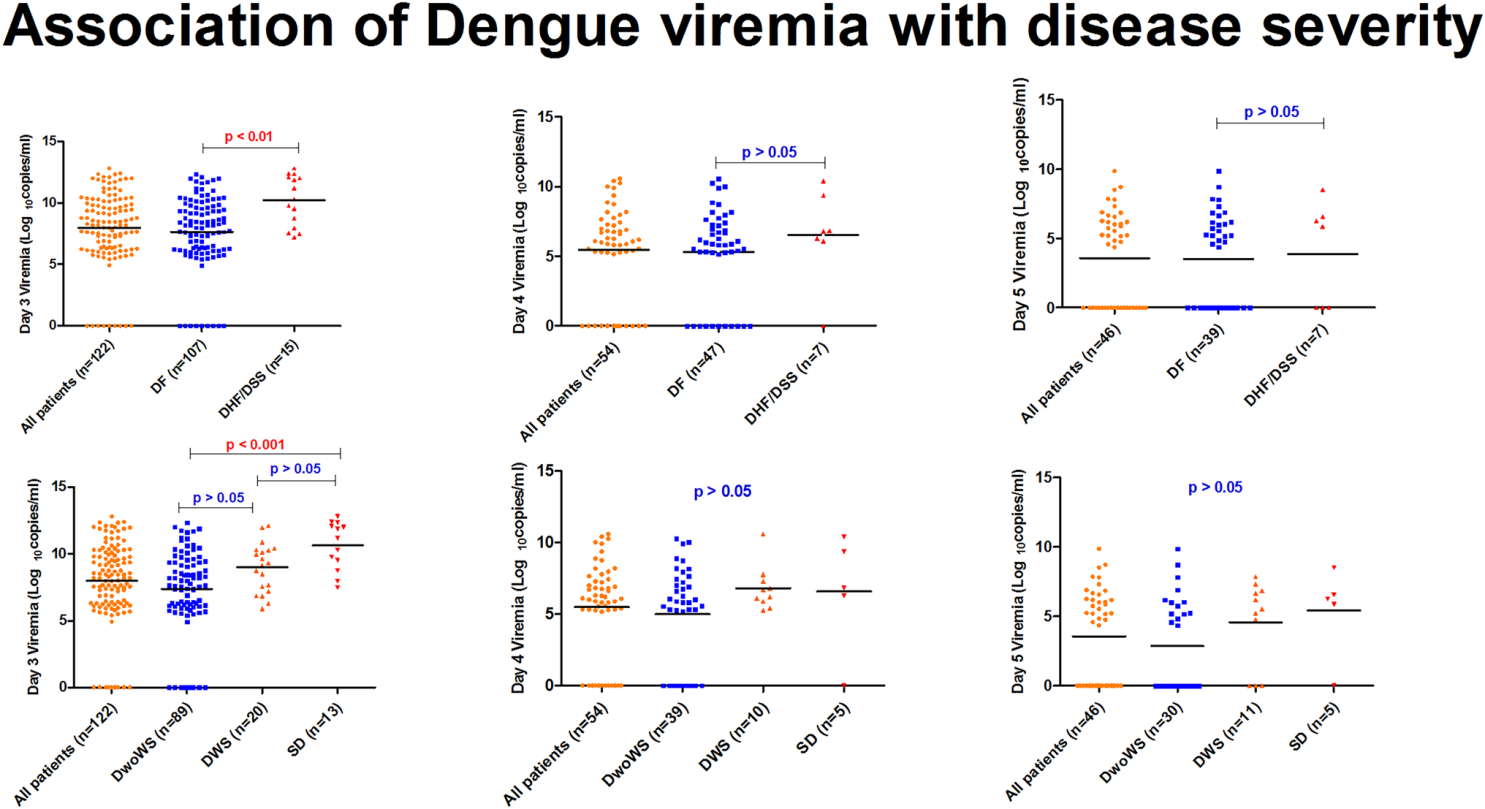
Association of dengue viremia with disease severity

Day wise viremia levels were plotted against the disease severity. It was observed that median viremia levels were higher for DHF/DSS patients as compared to DF patients. Moreover, it was also seen that according to the 2009 revised dengue case classification, the daily median viremia levels were higher in severe dengue as compared to DWS and DwoWS (Fig. 3). However, statistically significant association was seen only with day 3 viremia levels (Fig. 4). Moreover, there was no difference in mean time to viral clearance between DF and DHF/DSS (*p* value > 0.05).

**Fig. 5:**
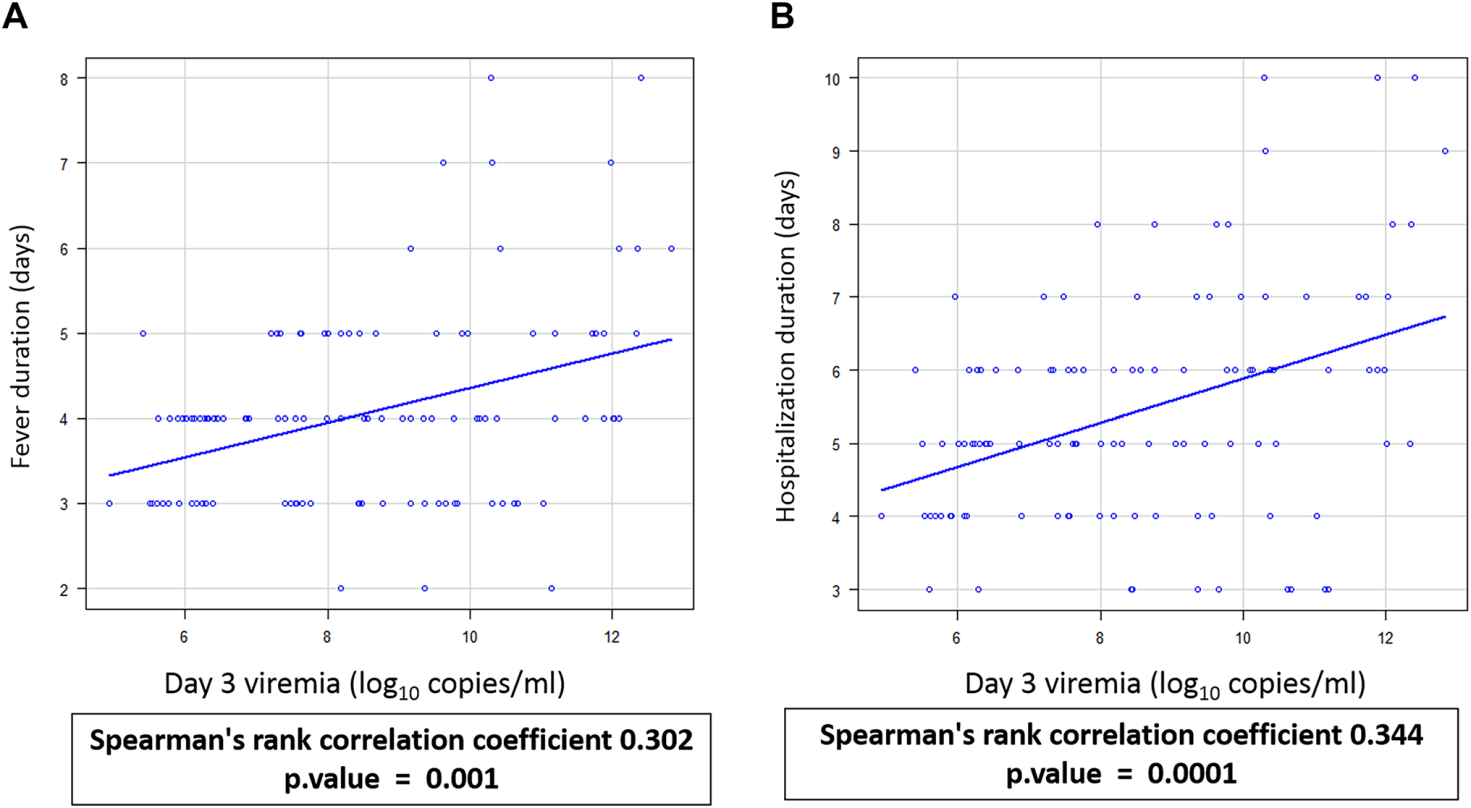
Association of day 3 dengue viremia levels with duration of fever and hospitalization. Each blue dot represents a sample, and the black line represents the linear relationship between the two continuous variables. **A** Association of day 3 viremia with duration of fever. **B** Association of day 3 viremia with duration of hospitalization

**Fig. 6:**
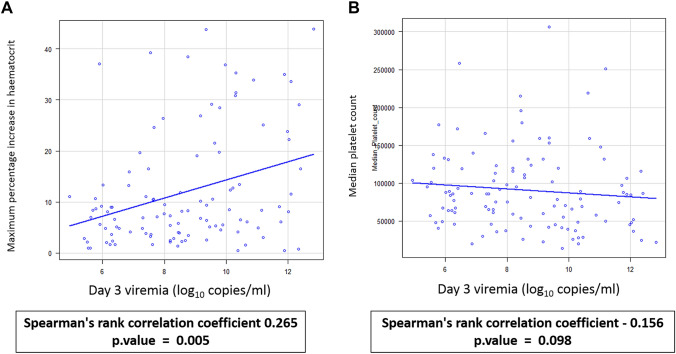
Association of day 3 viremia levels with median platelet count and maximum percentage increase in hematocrit. Each blue dot represents a sample, and the black line represents the linear relationship between the two continuous variables. **A** Association of day 3 viremia levels with maximum percentage increase in hematocrit. **B** Association of day 3 viremia levels with median platelet count

Moderate linear correlation was observed between day 3 viremia levels and duration of fever (Spearman’s rank correlation coefficient 0.344, *p* value < 0.001) and hospitalization (Spearman’s rank correlation coefficient 0.302, *p* value < 0.01) and is shown in Figure 5. Weak positive correlation was seen between day 3 viremia levels and maximum percentage increase in hematocrit (Spearman’s rank correlation coefficient 0.265, *p* value < 0.01). However, no statistically significant correlation was observed between day 3 viremia and median platelet count (Fig. 6).

### Day 3 viremia as a predictor of disease severity

**Fig. 7:**
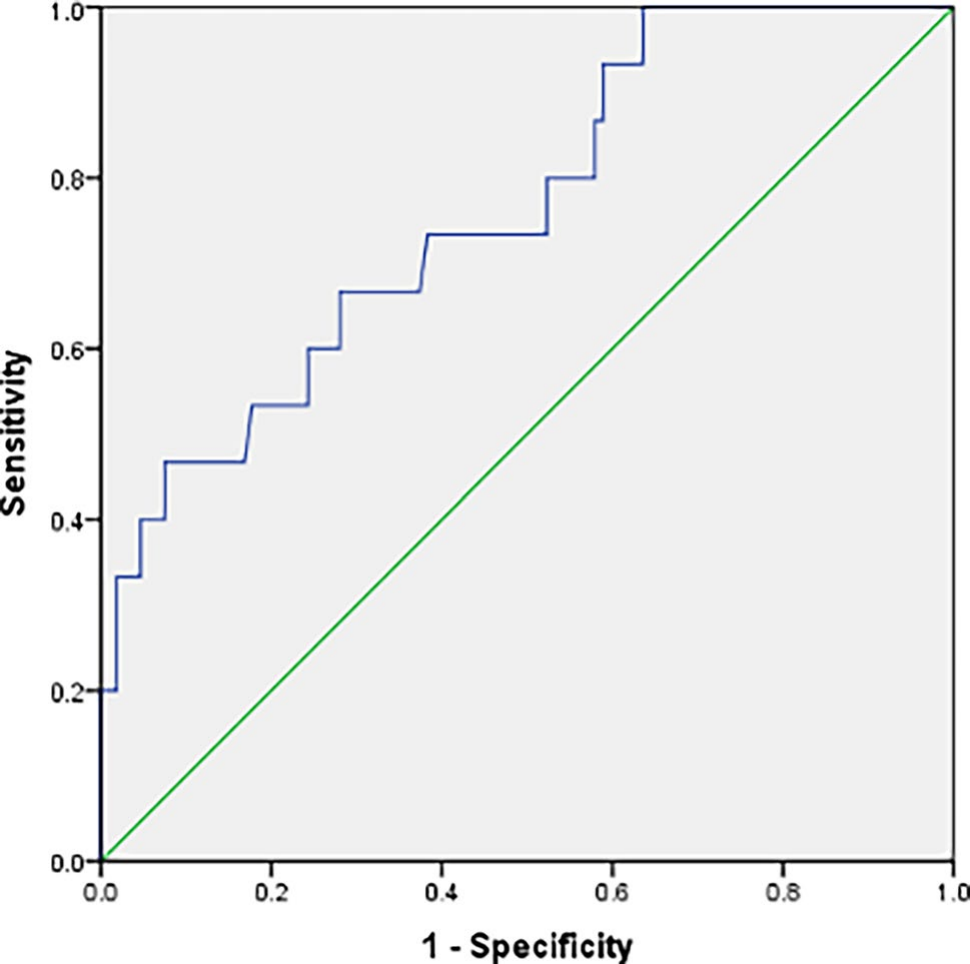
Receiver operating curve for evaluating Day 3 dengue viremia as a predictor of disease severity

Receiver operating characteristic (ROC) curve was used to evaluate the diagnostic capability of Day 3 viremia as a predictor of severe dengue. Interpretation of the area under the ROC curve showed that the performance of Day 3 viremia level (AUC 0.763; 95% CI: 0.635–0.890) was satisfactory. It was seen that a cut-off value of 7.055 log_10_ copies/ml of viremia proved to have a very high sensitivity of 100% but a poor specificity of 36.4% to predict severe dengue disease (Fig. 7). However, it was also seen that a cut-off value of 8.719 log_10_ copies/ml of viremia had a reasonable sensitivity of 73.3% and specificity of 61.7%.

## 4. Discussion

Despite extensive research in the epidemiology and pathogenesis of dengue virus infection, the identification of host or viral markers for early prediction of dengue disease severity has been never ending. Severe dengue disease and DHF/DSS remains a threat to millions of people across the globe. This threat is compounded by the fact that there is no specific treatment or vaccine available for the disease.

Real time qRT-PCR was performed to quantify the viremia levels in serum samples of laboratory confirmed dengue patients. To standardize the PCR, log dilutions of the oligonucleotide calibrator were prepared and made to run on QuantStudio 6 Flex Real-Time PCR system (Thermo Fisher Scientific, USA). The limit of detection in our study was found to be 40 copies/5 µL. This was much higher than the limit of detection described in the study by Gurukumar et al., who found their limit of detection to be 10 copies/0.5 µL, i.e. 1 copy/5 µL [6]. After standardization of the assay, qRT-PCR was performed with serum samples of dengue patients to quantify the viremia levels and later the values were log-transformed as log_10_ copies/ml. For statistical analysis, the samples in which viremia levels could not be detected were given an arbitrary value of one copy/ml, which after log conversion amounted to zero.

Conventional nested RT-PCR was performed for determination of the infecting serotype of dengue. The most common serotype observed in our study was Serotype-1 in 37 cases followed by serotype-2 in 34, serotype-3 in 10 and serotype-4 in 3 patients. It was observed that the conventional RT-PCR as described by Lanciotti et al., does not have a very high sensitivity as in 42 cases, the infecting serotype could not be detected due to low viral load.

In this study of 126 laboratory confirmed dengue cases, we used PanBio IgG capture ELISA in conjunction with PanBio IgM capture ELISA to differentiate between primary and secondary dengue and found 72 cases of primary dengue (57%) as compared to 54 cases of secondary dengue (43%). Similar technique was also used by Rao C et al., in their study and they also found a higher prevalence of primary dengue infections than secondary [11]. In our study we found that out of 54 secondary dengue cases, 14 were DHF/DSS or SD as compared to just one SD in primary dengue. It is a well-known fact that more severe disease tends to occur in secondary dengue infections due to antibody dependent enhancement due to heterologous antibody, which is not protective.

Viremia levels were quantified in serial daily samples of dengue patients of 60 patients and illness day 3 of 66 patients. Highest median viremia levels were observed for illness day 2 followed by illness day 3. As the infection progressed, the viremia levels kept on falling. However, none of these results were found to be statistically significant. However, de la Cruz-Hernandez et al., observed statistically significant decrease in viremia levels as the infection progressed [4]. Day-wise viremia levels were also compared between primary and secondary dengue patients and no significant difference was observed. This contrasts with the study by Vaughn DW et al., who found that rapid virus clearance occurred in secondary dengue as compared to primary dengue, which may be due to relatively rapid response of primed immune system in secondary dengue cases to clear the virus [13].

Viremia levels were also compared with the dengue serotypes. However, no significant association was found between day 3 viremia, peak viremia, time to viral clearance and dengue serotypes. We could compare only serotype 1 and 2 in our study because the serotype 3 and serotype 4 cases were too less for statistical analysis. However, Yung et al. observed higher dengue viral RNA levels in DENV-1 as compared to DENV-2 or DENV-3 [16]. Fox et al., also demonstrated that viral clearance was faster for DENV-2 than for DENV-1 patients [5].

Day wise viremia levels were compared between DF and DHF/DSS cases (WHO 1997 case classification) as well as DwoWS, DWS and SD patients (WHO revised 2009 case classification). Daily median viremia levels were observed to be higher in severe dengue and DHF/DSS cases as compared to DF. However, statistically significant difference was seen only for day 3 viremia levels, which showed significant association with dengue disease severity. Wang et al., in their study demonstrated that high levels of plasma RNA during defervescence (after fever subsides) had a significant association with disease severity and higher RNA levels at defervescence could serve as a marker for disease severity [14]. Similarly Vaughn et al., also showed that high viremia titers 3 days after illness onset correlated with disease severity [13].

In our study, we did not find any significant difference in the time to viral clearance between non-severe and severe dengue cases. Similar results were also obtained by Vaughn et al., who did not find any association between severity and duration of viremia [13].

In secondary dengue infections, it was found that day 3 viremia and peak viremia was significantly associated with disease severity and higher levels were seen in SD and DHF/DSS cases as compared to DF and DwoWS cases. This association could not be evaluated in primary dengue infections as only one cases of severe dengue was observed in primary dengue infections.

Platelet counts and increase in hematocrit have been used as laboratory indicators for development of severe disease and are used as one of the criteria to differentiate between DF and DHF/DSS cases in WHO 1997 dengue case classification and DwoWS, DWS and SD cases in WHO 2009 revised case classification [7, 15]. In the present study, weak negative correlation was observed between day 3 viremia and median platelet count, which was not statistically significant. We found a significant weak positive correlation between day 3 viremia levels and maximum percentage increase in hematocrit with correlation coefficient of 0.265. However, Alurdi et al., observed that dengue viral load had a significant inverse relationship with platelet count, with correlation coefficient of − 0.729. They also showed that a moderate positive linear relationship exists between dengue viral load and increase in hematocrit [1]. In this study, we also observed significant moderate positive linear relationship between day 3 viremia and duration of fever and hospitalization, with a correlation coefficient of 0.344 and 0.302 respectively.

In the present study, we evaluated the usefulness of dengue viremia for early prediction of severe dengue disease. ROC curve was constructed to assess the diagnostic ability of day 3 viremia. It was seen that a cut-off value of 7.055 log_10_ copies/ml of viremia proved to have a very high sensitivity of 100% but a poor specificity of 36.4% to predict severe dengue disease. However, it was also seen that a cut-off value of 8.719 log_10_ copies/ml of viremia had a reasonable sensitivity of 73.3% and specificity of 61.7%. In a study by Wang W-K et al., demonstrated that plasma dengue RNA levels higher than 10^4^ copies/ml at day 2 of defervescence was able to distinguish DHF from DF patients [14].

To the best of our knowledge and literature review, this is the first study of its kind in India which assesses the dengue viremia kinetics and its association with disease severity. Moreover, very few studies have evaluated the significance of dengue viremia levels in early disease as a predictor for severe dengue. Furthermore, this study also describes in detail the real time qRT-PCR for quantifying dengue viremia serum samples of laboratory confirmed dengue patients. However, this study has certain limitations. Firstly, not all the patients could be followed up prospectively and only 60 patients were followed by prospectively till discharge from the hospital and other 66 were included in the study retrospectively. Secondly, intra-serotypic differences in viremia levels could not be evaluated as there were very few cases of DENV-3 and DENV-4 infections for statistical analysis to be carried out.

## Conclusion

To conclude, the present study demonstrates a significant association between dengue viremia levels and disease severity. Cut-off value of 8.719 log_10_copies/ml for day 3 dengue viremia can be used to predict severe dengue disease with reasonable sensitivity and specificity. However, these results should be interpreted with caution and need further evaluation by prospective cohort studies with larger sample size.

## References

[1] Almurdi, Efrida, Dia Rofind Z, Plantika MJ. Relationship of viral load toward platelet count and hematocrit level in DENV-2 infection. J Med Sci. 2020;20(2):49–54. 10.3923/jms.2020.49.54

[2] Bhatt S, Gething PW, Brady OJ, Messina JP, Farlow AW, Moyes CL, et al. The global distribution and burden of dengue. Nature. 2013;496(7446):504–7. 23563266 10.1038/nature12060 PMC3651993

[3] Chaturvedi UC, Agarwal R, Elbishbishi EA, Mustafa AS. Cytokine cascade in dengue hemorrhagic fever: implications for pathogenesis. FEMS Immunol Med Microbiol. 2000;28(3):183–8. 10865168 10.1111/j.1574-695X.2000.tb01474.x

[4] de la Cruz-Hernández SI, Flores-Aguilar H, González-Mateos S, López-Martinez I, Alpuche-Aranda C, Ludert JE, et al. Determination of viremia and concentration of circulating nonstructural protein 1 in patients infected with dengue virus in Mexico. Am J Trop Med Hyg. 2013;88(3):446–54. 23339203 10.4269/ajtmh.12-0023 PMC3592523

[5] Fox A, Le NMH, Simmons CP, Wolbers M, Wertheim HFL, Pham TK, et al. Immunological and viral determinants of dengue severity in hospitalized adults in Ha Noi, Viet Nam. PLoS Negl Trop Dis. 2011;5(3):e967. 21390156 10.1371/journal.pntd.0000967 PMC3046970

[6] Gurukumar K, Priyadarshini D, Patil J, Bhagat A, Singh A, Shah P, et al. Development of real time PCR for detection and quantitation of Dengue Viruses. Virol J. 2009;23(6):10. 10.1186/1743-422X-6-10 PMC2651855 19166574

[7] Hadinegoro SRS. The revised WHO dengue case classification: does the system need to be modified? Paediatr Int Child Health. 2012;32(s1):33–8. 22668448 10.1179/2046904712Z.00000000052 PMC3381438

[8] Hawley WA, Reiter P, Copeland RS, Pumpuni CB, Craig GB. Aedes albopictus in North America: probable introduction in used tires from northern Asia. Science. 1987;236(4805):1114–6. 3576225 10.1126/science.3576225

[9] Lanciotti RS, Calisher CH, Gubler DJ, Chang GJ, Vorndam AV. Rapid detection and typing of dengue viruses from clinical samples by using reverse transcriptase-polymerase chain reaction. J Clin Microbiol. 1992;30(3):545–51. 1372617 10.1128/jcm.30.3.545-551.1992 PMC265106

[10] Mutheneni SR, Morse AP, Caminade C, Upadhyayula SM. Dengue burden in India: recent trends and importance of climatic parameters. Emerg Microbes Infect. 2017;6(8):e70. 28790459 10.1038/emi.2017.57 PMC5583666

[11] Rao C, Kaur H, Gupta N, Sabeena SP, Ambica R, Jain A, et al. Geographical distribution of primary and secondary dengue cases in India-2017: a cross-sectional multicentric study. Indian J Med Res. 2019;149(4):548–53. 31411180 10.4103/ijmr.IJMR_916_18 PMC6676848

[12] Special Programme for Research and Training in Tropical Diseases and World Health Organization. Dengue guidelines for diagnosis, treatment; 2009. preve.pdf [Internet]. Available from: http://www.who.int/tdr/publications/documents/dengue-diagnosis.pdf Accessed 03 Jul 2018.

[13] Vaughn DW, Green S, Kalayanarooj S, Innis BL, Nimmannitya S, Suntayakorn S, et al. Dengue viremia titer, antibody response pattern, and virus serotype correlate with disease severity. J Infect Dis. 2000;181(1):2–9. 10608744 10.1086/315215

[14] Wang WK, Chao DY, Kao CL, Wu HC, Liu YC, Li CM, et al. High levels of plasma dengue viral load during defervescence in patients with dengue hemorrhagic fever: implications for pathogenesis. Virology. 2003;305(2):330–8. 12573578 10.1006/viro.2002.1704

[15] World Health Organization. Dengue haemorrhagic fever diagnosis, treatment; 1997. p.pdf [Internet]. Available from: http://apps.who.int/iris/bitstream/handle/10665/41988/9241545003_eng.pdf;jsessionid=839B7D324058697247721C394B7C052F?sequence=1 Accessed 03 Jul 2018.

[16] Yung CF, Lee KS, Thein TL, Tan LK, Gan VC, Wong JGX, et al. Dengue serotype-specific differences in clinical manifestation, laboratory parameters and risk of severe disease in adults. Singap Am J Trop Med Hyg. 2015;92(5):999–1005. 10.4269/ajtmh.14-0628 PMC4426593 25825386

